# Methyl 4′-(3-bromo­phen­yl)-3′-(2,5-di­methyl­benz­yl)-1′-methyl-2-oxo­spiro­[indo­line-3,2′-pyrrolidine]-3′-carboxyl­ate

**DOI:** 10.1107/S1600536814002967

**Published:** 2014-02-15

**Authors:** S. Karthikeyan, K. Sethusankar, Anthonisamy Devaraj, Manickam Bakthadoss

**Affiliations:** a Department of Physics, RKM Vivekananda College (Autonomous), Chennai 600 004, India; b Department of Organic Chemistry, University of Madras, Maraimalai Campus, Chennai 600 025, India

## Abstract

In the title compound, C_29_H_29_BrN_2_O_3_, the indole ring system is essentially planar (r.m.s. deviation = 0.079 Å) and makes a dihedral angle of 85.23 (10)° with the mean plane of the 4-methyl­pyrrolidine ring. This ring adopts an envelope conformation with the N atom at the flap. The pyrrolidine ring of the indole ring system adopts a twisted conformation on the C—C(=O) bond. The mol­ecular structure is stabilized by an intra­molecular C—H⋯O hydrogen bond, which generates an *S*(6) ring motif. In the crystal, mol­ecules are linked *via* pairs of C—H⋯O hydrogen bonds, forming inversion dimers with an *R*_2_^2^(14) ring motif. These dimers are further linked by N—H⋯O and C—H⋯O hydrogen bonds, forming two-dimensional networks lying parallel to (10-1).

## Related literature   

For the biological activity of spiro-pyrrolidine derivatives, see: Obniska *et al.* (2002 ▶); Saito *et al.* (1991 ▶); Hilton *et al.* (2000 ▶). For related crystal structures, see: Jagadeesan *et al.* (2013 ▶). For puckering parameters, see: Cremer & Pople (1975 ▶). For graph-set motif notations, see: Bernstein *et al.* (1995 ▶). For bond-length distortions in small rings, see: Allen (1981 ▶).
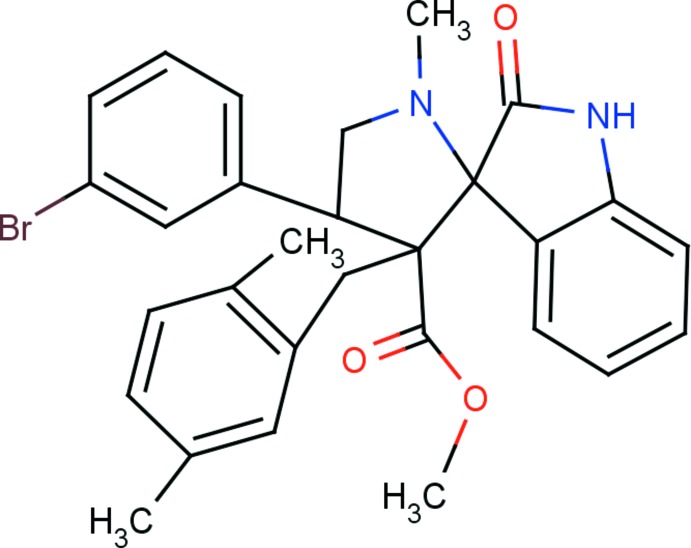


## Experimental   

### 

#### Crystal data  

C_29_H_29_BrN_2_O_3_
*M* *_r_* = 533.45
Monoclinic, 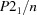

*a* = 12.0673 (4) Å
*b* = 9.4109 (3) Å
*c* = 22.5852 (7) Å
β = 103.660 (2)°
*V* = 2492.32 (14) Å^3^
*Z* = 4
Mo *K*α radiation
μ = 1.68 mm^−1^
*T* = 293 K
0.35 × 0.30 × 0.25 mm

#### Data collection  

Bruker Kappa APEXII CCD diffractometer
Absorption correction: multi-scan (*SADABS*; Bruker 2008 ▶) *T*_min_ = 0.564, *T*_max_ = 0.657
23085 measured reflections
4898 independent reflections
3487 reflections with *I* > 2σ(*I*)
*R*_int_ = 0.032

#### Refinement  

*R*[*F* ^2^ > 2σ(*F* ^2^)] = 0.035
*wR*(*F* ^2^) = 0.097
*S* = 1.01
4898 reflections
320 parameters
H-atom parameters constrained
Δρ_max_ = 0.28 e Å^−3^
Δρ_min_ = −0.37 e Å^−3^

### 

Data collection: *APEX2* (Bruker, 2008 ▶); cell refinement: *SAINT* (Bruker, 2008 ▶); data reduction: *SAINT*; program(s) used to solve structure: *SHELXS97* (Sheldrick, 2008 ▶); program(s) used to refine structure: *SHELXL97* (Sheldrick, 2008 ▶); molecular graphics: *ORTEP-3 for Windows* (Farrugia, 2012 ▶) and *Mercury* (Macrae *et al.*, 2008 ▶); software used to prepare material for publication: *SHELXL97* and *PLATON* (Spek, 2009 ▶).

## Supplementary Material

Crystal structure: contains datablock(s) global, I. DOI: 10.1107/S1600536814002967/su2698sup1.cif

Structure factors: contains datablock(s) I. DOI: 10.1107/S1600536814002967/su2698Isup2.hkl

Supporting information file. DOI: 10.1107/S1600536814002967/su2698Isup3.cml

CCDC reference:

Additional supporting information:  crystallographic information; 3D view; checkCIF report

## Figures and Tables

**Table 1:** Hydrogen-bond geometry (Å, °)

*D*—H⋯*A*	*D*—H	H⋯*A*	*D*⋯*A*	*D*—H⋯*A*
C5—H5⋯O1	0.93	2.39	3.253 (3)	155
N2—H2⋯O3^i^	0.86	2.24	3.085 (2)	166
C1—H1⋯O2^ii^	0.93	2.42	3.321 (2)	163
C26—H26*B*⋯O1^iii^	0.96	2.52	3.315 (3)	140

Symmetry codes: (i) 
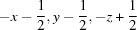
; (ii) 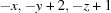; (iii) 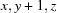.

**Fig. 1.**
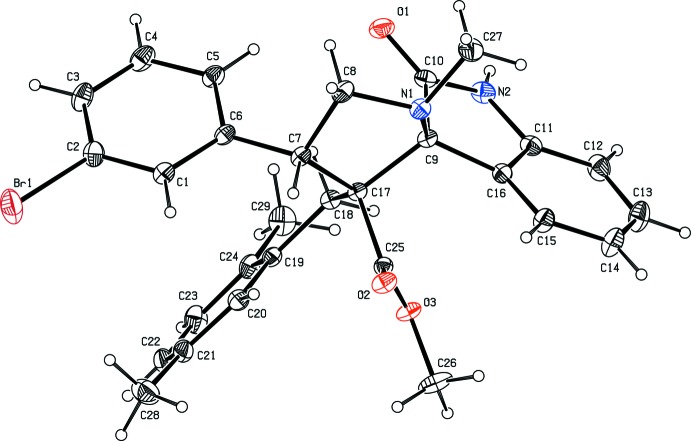
The molecular structure of the title comolecule, with atom labelling. Displacement ellipsoids are drawn at 30% probability level.

**Fig. 2.**
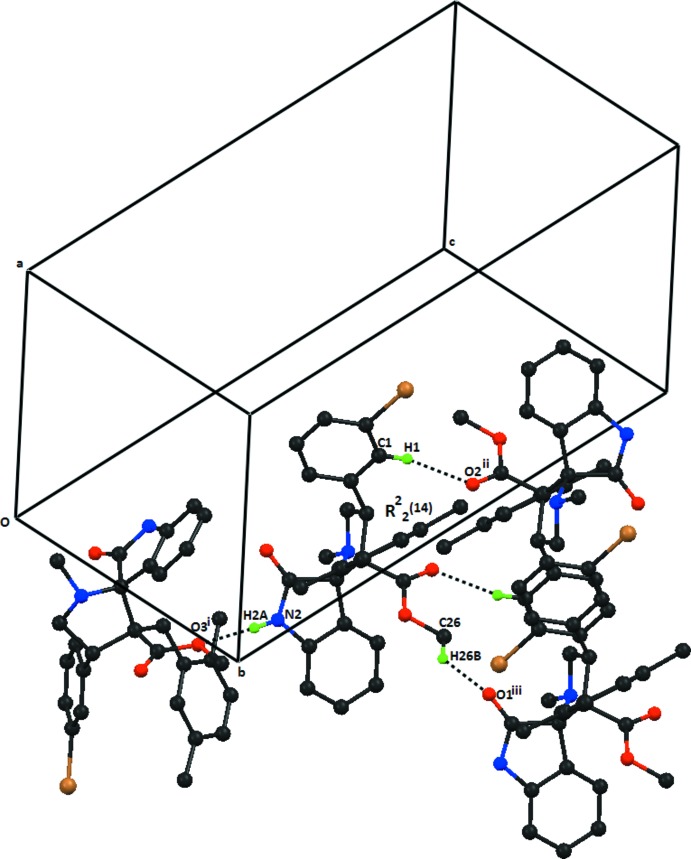
Aview of the crystal packing of the title compound, showing the formation of infinite chains C(7) and C(8) and R22(14) graph-set motifs. The dashed lines indicate hydrogen bonds (see Table 1 for details).

## supplementary crystallographic information

### 1. Comment

Spiro-compounds represent an important class of naturally occurring substances characterized by highly pronounced biological properties. Pyrrolidine derivatives posses anti-influenza virus and anti-convulsant activities (Obniska *et al.*, 2002; Hilton *et al.*, 2000). They also show inhibitory activity towards post-proline cleaving enzymes and show strong anti-amensic activities (Saito *et al.*, 1991).

The molecular structure of the title compound is illustrated in Fig 1. In the molecule, there is a C—H···O hydrogen bond, forming an *S*(6) ring motif (Table 1; Bernstein *et al.*, 1995). The indole ring system (N2/C9-C16) is essentially planar with a maximum deviation of -0.136 (2) Å for atom C10. Atom O1 deviates significantly from the mean plane of the indole ring system by -0.393 (2) Å. The mean plane of the indole ring system forms a dihedral angle of 85.23 (10)° with mean plane of the 4-methyl pyrrolidine ring (N1/C7-C9/C17). The mean plane of the 4-methyl pyrrolidine ring forms a dihedral angle of 52.81 (11)° with the benzyl ring. The molecular dimensions in the title compound are in excellent agreement with the corresponding values reported for a closely related compound (Jagadeesan *et al.*, 2013).

The spiro-pyrrolidine ring (N1/C7-C9/C17) adopts an envelope conformation with atom N1 at the flap. The distance to the flap position from the mean plane of the spiro carbon is 0.2455 (19) Å. The puckering parameters (Cremer & Pople, 1975) of the ring are Q_2_ = 0.390 (2) Å and φ_2_ = 174.9 (3)°. The pyrrolidine ring of the indole ring system adopts a twisted conformation on bond C9-C10, with deviations of -0.0621 (2) and 0.059 (2) Å, respectively, for the two atoms. The central spiro-pyrrolidine ring (N1/C7-C9/C17) is perpendicular to the bromophenyl ring with a dihedral angle of 88.32 (11)°. The carbonyl group and the benzyl ring have an (-)anti-periplanar conformation with torsion angle (C18—C17—C25—O2) being -154.57 (19)°.

In benzene ring (C11—C16) of the indole ring system, the expansion of the ipso angles at C11, C13 and C14 [121.8 (2), 121.3 (2) and 120.2 (2)°, respectively] and contraction of the apical angles at C12, C15 and C16 [117.9 (2), 119.1 (2) and 119.56 (19)°, respectively] are caused by the fusion of the smaller pyrrole ring to the six-membered benzene ring and the strain is taken up by the angular distortion rather than by bond-length distortions (Allen, 1981). The carboxyl group and oxindole ring system are (+)*syn*-clinal to each other with the torsion angle (C9—C17—C25—O2) of 84.5 (2)°.

The crystal packing (Fig. 2 and Table 1) is stabilized by the N2—H2···O3^i^ [symmetry code: (i) - *x*-1/2, + *y*-1/2, - *z*+1/2] hydrogen bond that generates C(7) chains, running parallel to the b axis. A second chain, C(8), running parallel to the same axis is formed by the C26—H26B···O1^iii^ [symmetry code: (iii) *x*, *y+1*, *z*] hydrogen bond. The molecules are further linked *via* C1—H1···O2^ii^ [symmetry code: (ii) -*x*, - *y+2*, - *z+1*] hydrogen bonds to form inversion dimers, resulting in *R*_2_^2^(14) graph-set motifs (Bernstein *et al.*, 1995). The combination of these various hydrogen bonds resuts in the formation of two-dimensional networks lying parallel to (101).

### 2. Experimental

A mixture of (*E*)-methyl 3-(3-bromophenyl)-2-(2,5-dimethylbenzyl)acrylate (2 mmol), isatin (2 mmol) and sarcosine (2 mmol) in acetonitrile (8 ml) was refluxed for 12 h. After the completion of the reaction as indicated by TLC, the reaction mixture was concentrated. The resulting crude mass was diluted with water (10 ml) and extracted with ethyl acetate (3 × 10 ml). The combined organic layers were washed with brine (2 × 10 ml) and dried over anhydrous Na_2_SO_4_. The organic layer was concentrated and the residue purified by column chromatography on silica gel (Acme 100–200 mesh), using ethyl acetate:hexanes (2:8) to afford the title compound as a colourless solid (Yield 71%). Block-like colourless crystals were obtained by slow evaporation of a solution in CHCl_3_.

### 3. Refinement

The H atoms could all be located in difference electron-density maps. In the final cycles of refinement they were treated as riding atoms and their distances were geometrically constrained: C—H = 0.93 and 0.96 Å for CH and CH_3_ H atoms, respectively, with *U*_iso_(H) = 1.5 *U*_eq_(C–methyl) and = 1.2*U*_eq_(C) for other H atoms.

### Crystal data

**Table:**

C_29_H_29_BrN_2_O_3_	*F*(000) = 1104
*M**_r_* = 533.45	*D*_x_ = 1.422 Mg m^−^^3^
Monoclinic, *P*2_1_/*n*	Mo *K*α radiation, λ = 0.71073 Å
Hall symbol: -p 2yn	Cell parameters from 4898 reflections
*a* = 12.0673 (4) Å	θ = 2.2–26.0°
*b* = 9.4109 (3) Å	µ = 1.68 mm^−^^1^
*c* = 22.5852 (7) Å	*T* = 293 K
β = 103.660 (2)°	Block, colorless
*V* = 2492.32 (14) Å^3^	0.35 × 0.30 × 0.25 mm
*Z* = 4	

### Data collection

**Table:**

Bruker Kappa APEXII CCD diffractometer	4898 independent reflections
Radiation source: fine-focus sealed tube	3487 reflections with *I* > 2σ(*I*)
Graphite monochromator	*R*_int_ = 0.032
ω scans	θ_max_ = 26.0°, θ_min_ = 2.2°
Absorption correction: multi-scan (*SADABS*; Bruker 2008)	*h* = −14→14
*T*_min_ = 0.564, *T*_max_ = 0.657	*k* = −11→11
23085 measured reflections	*l* = −27→23

### Refinement

**Table:**

Refinement on *F*^2^	Primary atom site location: structure-invariant direct methods
Least-squares matrix: full	Secondary atom site location: difference Fourier map
*R*[*F*^2^ > 2σ(*F*^2^)] = 0.035	Hydrogen site location: inferred from neighbouring sites
*wR*(*F*^2^) = 0.097	H-atom parameters constrained
*S* = 1.01	*w* = 1/[σ^2^(*F*_o_^2^) + (0.049*P*)^2^ + 0.846*P*] where *P* = (*F*_o_^2^ + 2*F*_c_^2^)/3
4898 reflections	(Δ/σ)_max_ = 0.001
320 parameters	Δρ_max_ = 0.28 e Å^−^^3^
0 restraints	Δρ_min_ = −0.37 e Å^−^^3^

### Special details

**Table:**

Geometry. All e.s.d.'s (except the e.s.d. in the dihedral angle between two l.s. planes) are estimated using the full covariance matrix. The cell e.s.d.'s are taken into account individually in the estimation of e.s.d.'s in distances, angles and torsion angles; correlations between e.s.d.'s in cell parameters are only used when they are defined by crystal symmetry. An approximate (isotropic) treatment of cell e.s.d.'s is used for estimating e.s.d.'s involving l.s. planes.
Refinement. Refinement of *F*^2^ against ALL reflections. The weighted *R*-factor *wR* and goodness of fit *S* are based on *F*^2^, conventional *R*-factors *R* are based on *F*, with *F* set to zero for negative *F*^2^. The threshold expression of *F*^2^ > σ(*F*^2^) is used only for calculating *R*-factors(gt) *etc*. and is not relevant to the choice of reflections for refinement. *R*-factors based on *F*^2^ are statistically about twice as large as those based on *F*, and *R*- factors based on ALL data will be even larger.

### Fractional atomic coordinates and isotropic or equivalent isotropic displacement parameters (Å^2^)

**Table:**

	*x*	*y*	*z*	*U*_iso_*/*U*_eq_	
C1	0.19054 (18)	0.7464 (2)	0.45559 (9)	0.0337 (5)	
H1	0.1890	0.8197	0.4830	0.040*	
C2	0.29340 (19)	0.6963 (3)	0.44773 (10)	0.0406 (5)	
C3	0.2988 (2)	0.5886 (3)	0.40750 (11)	0.0480 (6)	
H3	0.3687	0.5564	0.4022	0.058*	
C4	0.1991 (2)	0.5298 (3)	0.37549 (10)	0.0469 (6)	
H4	0.2015	0.4563	0.3483	0.056*	
C5	0.09473 (19)	0.5775 (2)	0.38276 (10)	0.0376 (5)	
H5	0.0278	0.5356	0.3608	0.045*	
C6	0.08958 (17)	0.6878 (2)	0.42272 (9)	0.0309 (5)	
C7	−0.02180 (17)	0.7436 (2)	0.43366 (9)	0.0291 (5)	
H7	−0.0019	0.8094	0.4682	0.035*	
C8	−0.09576 (17)	0.6270 (2)	0.45184 (10)	0.0355 (5)	
H8A	−0.0850	0.5375	0.4327	0.043*	
H8B	−0.0772	0.6141	0.4957	0.043*	
C9	−0.22078 (17)	0.7398 (2)	0.37089 (9)	0.0269 (4)	
C10	−0.23087 (17)	0.6300 (2)	0.31808 (9)	0.0317 (5)	
C11	−0.38171 (18)	0.7809 (2)	0.29012 (9)	0.0333 (5)	
C12	−0.48250 (19)	0.8392 (3)	0.25872 (11)	0.0466 (6)	
H12	−0.5152	0.8125	0.2188	0.056*	
C13	−0.5336 (2)	0.9379 (3)	0.28800 (12)	0.0574 (7)	
H13	−0.6017	0.9796	0.2674	0.069*	
C14	−0.4859 (2)	0.9770 (3)	0.34747 (12)	0.0541 (7)	
H14	−0.5230	1.0426	0.3668	0.065*	
C15	−0.38347 (18)	0.9189 (2)	0.37835 (10)	0.0399 (5)	
H15	−0.3514	0.9444	0.4185	0.048*	
C16	−0.32970 (16)	0.8232 (2)	0.34905 (9)	0.0297 (5)	
C17	−0.10458 (16)	0.8266 (2)	0.38018 (8)	0.0258 (4)	
C18	−0.06611 (16)	0.8413 (2)	0.32054 (9)	0.0288 (4)	
H18A	−0.0465	0.7473	0.3087	0.035*	
H18B	−0.1310	0.8737	0.2894	0.035*	
C19	0.03291 (16)	0.9389 (2)	0.31960 (9)	0.0300 (5)	
C20	0.09418 (17)	1.0100 (2)	0.37079 (9)	0.0337 (5)	
H20	0.0720	0.9991	0.4072	0.040*	
C21	0.18680 (17)	1.0964 (2)	0.37017 (10)	0.0388 (5)	
C22	0.21530 (19)	1.1154 (3)	0.31553 (12)	0.0463 (6)	
H22	0.2756	1.1751	0.3134	0.056*	
C23	0.15609 (19)	1.0475 (3)	0.26388 (11)	0.0476 (6)	
H23	0.1772	1.0626	0.2274	0.057*	
C24	0.06569 (18)	0.9570 (2)	0.26467 (10)	0.0373 (5)	
C25	−0.13018 (16)	0.9724 (2)	0.40407 (9)	0.0283 (4)	
C26	−0.1905 (3)	1.2069 (2)	0.37822 (12)	0.0588 (8)	
H26A	−0.2565	1.2010	0.3949	0.088*	
H26B	−0.2058	1.2699	0.3438	0.088*	
H26C	−0.1272	1.2426	0.4087	0.088*	
C27	−0.2983 (2)	0.5739 (3)	0.43525 (11)	0.0453 (6)	
H27A	−0.3725	0.6139	0.4191	0.068*	
H27B	−0.2906	0.5493	0.4773	0.068*	
H27C	−0.2892	0.4902	0.4126	0.068*	
C28	0.2541 (2)	1.1619 (3)	0.42803 (12)	0.0599 (7)	
H28A	0.2029	1.1957	0.4516	0.090*	
H28B	0.2980	1.2400	0.4185	0.090*	
H28C	0.3044	1.0921	0.4510	0.090*	
C29	0.0049 (2)	0.8811 (3)	0.20775 (11)	0.0535 (7)	
H29A	0.0399	0.9050	0.1750	0.080*	
H29B	−0.0737	0.9095	0.1972	0.080*	
H29C	0.0098	0.7804	0.2146	0.080*	
N1	−0.21158 (14)	0.67732 (18)	0.43036 (7)	0.0313 (4)	
N2	−0.31885 (15)	0.67136 (19)	0.27236 (8)	0.0379 (4)	
H2	−0.3343	0.6344	0.2365	0.045*	
O1	−0.17294 (13)	0.52493 (16)	0.31804 (7)	0.0436 (4)	
O2	−0.12565 (12)	0.99948 (15)	0.45631 (6)	0.0369 (4)	
O3	−0.16324 (12)	1.06736 (14)	0.35912 (6)	0.0355 (3)	
Br1	0.43004 (2)	0.77598 (4)	0.494958 (15)	0.07456 (14)	

### Atomic displacement parameters (Å^2^)

**Table:**

	*U*^11^	*U*^22^	*U*^33^	*U*^12^	*U*^13^	*U*^23^
C1	0.0376 (11)	0.0362 (12)	0.0262 (11)	0.0051 (9)	0.0051 (9)	−0.0006 (9)
C2	0.0361 (11)	0.0516 (15)	0.0331 (12)	0.0029 (10)	0.0063 (10)	0.0019 (11)
C3	0.0414 (13)	0.0620 (17)	0.0441 (14)	0.0161 (11)	0.0170 (11)	−0.0023 (12)
C4	0.0540 (14)	0.0501 (15)	0.0366 (13)	0.0154 (12)	0.0109 (11)	−0.0081 (11)
C5	0.0406 (12)	0.0369 (13)	0.0325 (12)	0.0075 (10)	0.0030 (9)	−0.0042 (10)
C6	0.0358 (11)	0.0312 (12)	0.0245 (11)	0.0081 (9)	0.0050 (9)	0.0050 (9)
C7	0.0332 (10)	0.0304 (12)	0.0222 (10)	0.0068 (8)	0.0033 (8)	−0.0008 (8)
C8	0.0415 (12)	0.0361 (12)	0.0278 (11)	0.0097 (10)	0.0059 (9)	0.0098 (9)
C9	0.0327 (10)	0.0252 (11)	0.0225 (10)	0.0022 (8)	0.0058 (8)	−0.0001 (8)
C10	0.0385 (11)	0.0259 (11)	0.0317 (11)	−0.0047 (9)	0.0102 (9)	0.0002 (9)
C11	0.0373 (11)	0.0323 (12)	0.0290 (11)	−0.0044 (9)	0.0050 (9)	0.0034 (9)
C12	0.0398 (12)	0.0566 (16)	0.0360 (13)	−0.0027 (11)	−0.0060 (10)	0.0053 (12)
C13	0.0387 (13)	0.0726 (19)	0.0536 (17)	0.0163 (13)	−0.0033 (12)	0.0064 (14)
C14	0.0442 (13)	0.0628 (17)	0.0547 (16)	0.0201 (12)	0.0105 (12)	−0.0009 (13)
C15	0.0373 (11)	0.0454 (14)	0.0359 (12)	0.0070 (10)	0.0068 (10)	−0.0032 (11)
C16	0.0306 (10)	0.0305 (11)	0.0273 (11)	−0.0007 (9)	0.0052 (8)	0.0027 (9)
C17	0.0309 (10)	0.0249 (10)	0.0202 (10)	0.0025 (8)	0.0032 (8)	−0.0009 (8)
C18	0.0346 (10)	0.0287 (11)	0.0228 (10)	0.0019 (9)	0.0064 (8)	−0.0005 (8)
C19	0.0318 (10)	0.0288 (11)	0.0292 (11)	0.0054 (9)	0.0068 (9)	0.0048 (9)
C20	0.0358 (11)	0.0343 (12)	0.0303 (11)	0.0025 (9)	0.0066 (9)	0.0027 (10)
C21	0.0315 (11)	0.0390 (13)	0.0438 (13)	0.0021 (9)	0.0049 (10)	0.0065 (11)
C22	0.0309 (11)	0.0473 (15)	0.0623 (16)	−0.0012 (10)	0.0142 (11)	0.0078 (13)
C23	0.0432 (13)	0.0630 (17)	0.0424 (14)	0.0029 (12)	0.0220 (11)	0.0072 (12)
C24	0.0365 (11)	0.0448 (13)	0.0323 (12)	0.0083 (10)	0.0114 (9)	0.0018 (10)
C25	0.0286 (10)	0.0273 (11)	0.0278 (12)	0.0012 (8)	0.0043 (8)	0.0003 (9)
C26	0.097 (2)	0.0291 (14)	0.0526 (16)	0.0220 (14)	0.0221 (15)	0.0055 (12)
C27	0.0494 (13)	0.0448 (14)	0.0441 (14)	−0.0038 (11)	0.0158 (11)	0.0099 (11)
C28	0.0589 (16)	0.0549 (17)	0.0590 (17)	−0.0165 (13)	0.0001 (13)	−0.0031 (13)
C29	0.0605 (15)	0.0702 (19)	0.0335 (13)	0.0021 (14)	0.0187 (12)	−0.0041 (12)
N1	0.0363 (9)	0.0324 (10)	0.0255 (9)	0.0027 (8)	0.0082 (7)	0.0065 (7)
N2	0.0466 (10)	0.0388 (11)	0.0244 (9)	−0.0049 (9)	0.0009 (8)	−0.0063 (8)
O1	0.0519 (9)	0.0305 (9)	0.0476 (10)	0.0035 (7)	0.0101 (8)	−0.0076 (7)
O2	0.0509 (9)	0.0340 (8)	0.0241 (8)	0.0075 (7)	0.0058 (6)	−0.0052 (6)
O3	0.0515 (9)	0.0246 (8)	0.0305 (8)	0.0110 (7)	0.0098 (7)	0.0047 (6)
Br1	0.03672 (15)	0.1020 (3)	0.0809 (2)	−0.00654 (14)	0.00577 (14)	−0.02329 (18)

### Geometric parameters (Å, º)

**Table:**

C1—C2	1.378 (3)	C15—H15	0.9300
C1—C6	1.383 (3)	C17—C18	1.531 (3)
C1—H1	0.9300	C17—C25	1.532 (3)
C2—C3	1.373 (3)	C18—C19	1.511 (3)
C2—Br1	1.895 (2)	C18—H18A	0.9700
C3—C4	1.366 (3)	C18—H18B	0.9700
C3—H3	0.9300	C19—C20	1.388 (3)
C4—C5	1.383 (3)	C19—C24	1.399 (3)
C4—H4	0.9300	C20—C21	1.385 (3)
C5—C6	1.386 (3)	C20—H20	0.9300
C5—H5	0.9300	C21—C22	1.369 (3)
C6—C7	1.517 (3)	C21—C28	1.499 (3)
C7—C8	1.531 (3)	C22—C23	1.373 (3)
C7—C17	1.580 (3)	C22—H22	0.9300
C7—H7	0.9800	C23—C24	1.387 (3)
C8—N1	1.447 (3)	C23—H23	0.9300
C8—H8A	0.9700	C24—C29	1.502 (3)
C8—H8B	0.9700	C25—O2	1.196 (2)
C9—N1	1.446 (2)	C25—O3	1.341 (2)
C9—C16	1.510 (3)	C26—O3	1.444 (3)
C9—C10	1.561 (3)	C26—H26A	0.9600
C9—C17	1.593 (3)	C26—H26B	0.9600
C10—O1	1.211 (2)	C26—H26C	0.9600
C10—N2	1.352 (3)	C27—N1	1.452 (3)
C11—C12	1.370 (3)	C27—H27A	0.9600
C11—C16	1.389 (3)	C27—H27B	0.9600
C11—N2	1.394 (3)	C27—H27C	0.9600
C12—C13	1.369 (4)	C28—H28A	0.9600
C12—H12	0.9300	C28—H28B	0.9600
C13—C14	1.380 (4)	C28—H28C	0.9600
C13—H13	0.9300	C29—H29A	0.9600
C14—C15	1.381 (3)	C29—H29B	0.9600
C14—H14	0.9300	C29—H29C	0.9600
C15—C16	1.368 (3)	N2—H2	0.8600
			
C2—C1—C6	120.0 (2)	C25—C17—C9	105.13 (15)
C2—C1—H1	120.0	C7—C17—C9	103.01 (15)
C6—C1—H1	120.0	C19—C18—C17	118.18 (16)
C3—C2—C1	121.5 (2)	C19—C18—H18A	107.8
C3—C2—Br1	119.58 (17)	C17—C18—H18A	107.8
C1—C2—Br1	118.86 (17)	C19—C18—H18B	107.8
C4—C3—C2	118.4 (2)	C17—C18—H18B	107.8
C4—C3—H3	120.8	H18A—C18—H18B	107.1
C2—C3—H3	120.8	C20—C19—C24	118.23 (19)
C3—C4—C5	121.2 (2)	C20—C19—C18	123.28 (18)
C3—C4—H4	119.4	C24—C19—C18	118.48 (18)
C5—C4—H4	119.4	C21—C20—C19	123.2 (2)
C4—C5—C6	120.2 (2)	C21—C20—H20	118.4
C4—C5—H5	119.9	C19—C20—H20	118.4
C6—C5—H5	119.9	C22—C21—C20	117.5 (2)
C1—C6—C5	118.60 (19)	C22—C21—C28	122.2 (2)
C1—C6—C7	118.46 (18)	C20—C21—C28	120.3 (2)
C5—C6—C7	122.91 (19)	C21—C22—C23	120.9 (2)
C6—C7—C8	112.97 (17)	C21—C22—H22	119.5
C6—C7—C17	118.13 (16)	C23—C22—H22	119.5
C8—C7—C17	104.84 (16)	C22—C23—C24	121.8 (2)
C6—C7—H7	106.8	C22—C23—H23	119.1
C8—C7—H7	106.8	C24—C23—H23	119.1
C17—C7—H7	106.8	C23—C24—C19	118.3 (2)
N1—C8—C7	104.69 (16)	C23—C24—C29	120.5 (2)
N1—C8—H8A	110.8	C19—C24—C29	121.2 (2)
C7—C8—H8A	110.8	O2—C25—O3	122.75 (18)
N1—C8—H8B	110.8	O2—C25—C17	124.94 (18)
C7—C8—H8B	110.8	O3—C25—C17	112.27 (16)
H8A—C8—H8B	108.9	O3—C26—H26A	109.5
N1—C9—C16	112.63 (16)	O3—C26—H26B	109.5
N1—C9—C10	114.52 (16)	H26A—C26—H26B	109.5
C16—C9—C10	100.93 (15)	O3—C26—H26C	109.5
N1—C9—C17	102.36 (15)	H26A—C26—H26C	109.5
C16—C9—C17	116.60 (16)	H26B—C26—H26C	109.5
C10—C9—C17	110.34 (15)	N1—C27—H27A	109.5
O1—C10—N2	125.7 (2)	N1—C27—H27B	109.5
O1—C10—C9	126.89 (18)	H27A—C27—H27B	109.5
N2—C10—C9	107.35 (17)	N1—C27—H27C	109.5
C12—C11—C16	121.8 (2)	H27A—C27—H27C	109.5
C12—C11—N2	128.6 (2)	H27B—C27—H27C	109.5
C16—C11—N2	109.52 (18)	C21—C28—H28A	109.5
C13—C12—C11	117.9 (2)	C21—C28—H28B	109.5
C13—C12—H12	121.1	H28A—C28—H28B	109.5
C11—C12—H12	121.1	C21—C28—H28C	109.5
C12—C13—C14	121.3 (2)	H28A—C28—H28C	109.5
C12—C13—H13	119.4	H28B—C28—H28C	109.5
C14—C13—H13	119.4	C24—C29—H29A	109.5
C13—C14—C15	120.2 (2)	C24—C29—H29B	109.5
C13—C14—H14	119.9	H29A—C29—H29B	109.5
C15—C14—H14	119.9	C24—C29—H29C	109.5
C16—C15—C14	119.1 (2)	H29A—C29—H29C	109.5
C16—C15—H15	120.4	H29B—C29—H29C	109.5
C14—C15—H15	120.4	C8—N1—C9	107.51 (16)
C15—C16—C11	119.56 (19)	C8—N1—C27	114.32 (17)
C15—C16—C9	131.25 (18)	C9—N1—C27	116.39 (16)
C11—C16—C9	109.01 (18)	C10—N2—C11	112.08 (17)
C18—C17—C25	110.94 (16)	C10—N2—H2	124.0
C18—C17—C7	116.66 (16)	C11—N2—H2	124.0
C25—C17—C7	108.48 (15)	C25—O3—C26	115.35 (17)
C18—C17—C9	111.76 (15)		
			
C6—C1—C2—C3	0.2 (3)	C16—C9—C17—C18	−84.5 (2)
C6—C1—C2—Br1	−178.49 (16)	C10—C9—C17—C18	29.8 (2)
C1—C2—C3—C4	−0.8 (4)	N1—C9—C17—C25	−87.50 (17)
Br1—C2—C3—C4	177.86 (19)	C16—C9—C17—C25	35.9 (2)
C2—C3—C4—C5	0.5 (4)	C10—C9—C17—C25	150.20 (16)
C3—C4—C5—C6	0.5 (4)	N1—C9—C17—C7	26.04 (18)
C2—C1—C6—C5	0.8 (3)	C16—C9—C17—C7	149.43 (17)
C2—C1—C6—C7	178.69 (19)	C10—C9—C17—C7	−96.25 (17)
C4—C5—C6—C1	−1.1 (3)	C25—C17—C18—C19	54.2 (2)
C4—C5—C6—C7	−179.0 (2)	C7—C17—C18—C19	−70.7 (2)
C1—C6—C7—C8	−123.9 (2)	C9—C17—C18—C19	171.14 (16)
C5—C6—C7—C8	53.9 (3)	C17—C18—C19—C20	3.4 (3)
C1—C6—C7—C17	113.3 (2)	C17—C18—C19—C24	−177.57 (18)
C5—C6—C7—C17	−68.9 (3)	C24—C19—C20—C21	−0.8 (3)
C6—C7—C8—N1	−151.54 (17)	C18—C19—C20—C21	178.23 (19)
C17—C7—C8—N1	−21.6 (2)	C19—C20—C21—C22	2.5 (3)
N1—C9—C10—O1	−46.8 (3)	C19—C20—C21—C28	−175.7 (2)
C16—C9—C10—O1	−168.0 (2)	C20—C21—C22—C23	−1.9 (3)
C17—C9—C10—O1	68.0 (3)	C28—C21—C22—C23	176.2 (2)
N1—C9—C10—N2	131.64 (18)	C21—C22—C23—C24	−0.2 (4)
C16—C9—C10—N2	10.4 (2)	C22—C23—C24—C19	1.9 (3)
C17—C9—C10—N2	−113.53 (18)	C22—C23—C24—C29	−178.2 (2)
C16—C11—C12—C13	−2.3 (4)	C20—C19—C24—C23	−1.4 (3)
N2—C11—C12—C13	174.2 (2)	C18—C19—C24—C23	179.52 (19)
C11—C12—C13—C14	−0.6 (4)	C20—C19—C24—C29	178.7 (2)
C12—C13—C14—C15	1.5 (4)	C18—C19—C24—C29	−0.4 (3)
C13—C14—C15—C16	0.5 (4)	C18—C17—C25—O2	−154.57 (19)
C14—C15—C16—C11	−3.3 (3)	C7—C17—C25—O2	−25.2 (3)
C14—C15—C16—C9	−177.9 (2)	C9—C17—C25—O2	84.5 (2)
C12—C11—C16—C15	4.3 (3)	C18—C17—C25—O3	27.9 (2)
N2—C11—C16—C15	−172.80 (19)	C7—C17—C25—O3	157.27 (16)
C12—C11—C16—C9	−180.0 (2)	C9—C17—C25—O3	−93.08 (18)
N2—C11—C16—C9	2.9 (2)	C7—C8—N1—C9	41.1 (2)
N1—C9—C16—C15	44.6 (3)	C7—C8—N1—C27	171.93 (17)
C10—C9—C16—C15	167.2 (2)	C16—C9—N1—C8	−168.11 (17)
C17—C9—C16—C15	−73.3 (3)	C10—C9—N1—C8	77.3 (2)
N1—C9—C16—C11	−130.45 (18)	C17—C9—N1—C8	−42.09 (19)
C10—C9—C16—C11	−7.9 (2)	C16—C9—N1—C27	62.2 (2)
C17—C9—C16—C11	111.63 (19)	C10—C9—N1—C27	−52.4 (2)
C6—C7—C17—C18	1.2 (3)	C17—C9—N1—C27	−171.78 (17)
C8—C7—C17—C18	−125.60 (18)	O1—C10—N2—C11	168.9 (2)
C6—C7—C17—C25	−124.90 (19)	C9—C10—N2—C11	−9.6 (2)
C8—C7—C17—C25	108.28 (18)	C12—C11—N2—C10	−172.4 (2)
C6—C7—C17—C9	124.02 (18)	C16—C11—N2—C10	4.5 (3)
C8—C7—C17—C9	−2.80 (19)	O2—C25—O3—C26	1.5 (3)
N1—C9—C17—C18	152.06 (16)	C17—C25—O3—C26	179.14 (19)

### Hydrogen-bond geometry (Å, º)

**Table:**

*D*—H···*A*	*D*—H	H···*A*	*D*···*A*	*D*—H···*A*
C5—H5···O1	0.93	2.39	3.253 (3)	155
N2—H2···O3^i^	0.86	2.24	3.085 (2)	166
C1—H1···O2^ii^	0.93	2.42	3.321 (2)	163
C26—H26*B*···O1^iii^	0.96	2.52	3.315 (3)	140

Symmetry codes: (i) −*x*−1/2, *y*−1/2, −*z*+1/2; (ii) −*x*, −*y*+2, −*z*+1; (iii) *x*, *y*+1, *z*.
